# Hierarchical Control of an Integrated Fuel Processing and Fuel Cell System

**DOI:** 10.3390/ma12010021

**Published:** 2018-12-21

**Authors:** Markku Ohenoja, Mika Ruusunen, Kauko Leiviskä

**Affiliations:** Control Engineering, University of Oulu, P.O. Box 4300, FI-90014 Oulu, Finland; mika.ruusunen@oulu.fi (M.R.); kauko.leiviska@oulu.fi (K.L.)

**Keywords:** advanced control, model-based control, differential evolution, steam reforming, hydrogen purification, proton exchange membrane

## Abstract

An advanced model-based control method for the integrated fuel processing and a fuel cell system consisting of ethanol reforming, hydrogen purification, and a proton exchange membrane fuel cell is presented. For process identification, a physical model of the process chain was constructed. Subsequently, the simulated process was approximated with data-driven control models. Based on these control models, a hierarchical control framework consisting of model predictive controller and a global optimization algorithm was introduced. The performance of the new control method was evaluated with simulations. Results indicate that the new optimization concept enables resource efficient and fast control of the studied energy conversion process. Fast and efficient fuel cell process could then provide sustainable power source for autonomous and mobile applications in the future.

## 1. Introduction 

The energy sector is continuously aiming at more sustainable methods, saving the environment and fuels. The future energy production will be decentralized involving variety of energy conversion methods, energy storage capabilities, and smart grids. Fuel Cell (FC) technology is one key alternative to the existing technology burning fossil fuels. Its popularity in distributed energy systems origins from high efficiency, low risk to the environment, and flexibility in different applications [1]. The Proton Exchange Membrane (PEM) fuel cell has been found to be suitable for both residential and mobile applications, because it can operate at relatively low temperatures, it has relatively high power density and its maintenance is simple [2]. However, it requires a reliable source of pure hydrogen. Therefore, in situ fuel production from liquid hydrocarbons, such as methanol and ethanol, or from renewable sources, like biomasses, is preferable in autonomous fuel cell power systems.

There are several well-known techniques for hydrogen production, such as steam reforming, partial oxidation, and autothermal reforming [3]. Despite the conversion technique, also a purification stage is required prior to the fuel cell. Hence, the integrated fuel processing and fuel cell system consists of several unit processes targeted for hydrogen production, syngas cleaning and fuel cell system. Examples of possible process routes can be found from [4,5]. The overall efficiency increases with membrane technologies [6,7,8,9,10].

Apart from process design, the operational issues of the integrated systems may have a substantial effect on the overall efficiency. Three major objectives for the process control in such systems have been identified [11]: (1) to avoid fuel cell hydrogen starvation, (2) to keep reaction temperatures close to set-points, and (3) ensure high overall system efficiency. Conventional approaches with Single-Input Single-Output (SISO) and Multiple-Input Multiple-Output (MIMO) systems and standard Proportional-Integral (PI) controllers have been considered for the control problem [11,12,13,14,15,16,17]. The main challenge preventing the abovementioned objectives arises from the time delay between the hydrogen demand and supply. To avoid stack hydrogen starvation, a rapid and robust response after the set-point changes in the stack current is required [4]. Otherwise, the stack might be permanently damaged. As noted in the same reference, the oversupply of hydrogen easily leads to fuel losses. Compensation for this delay requires model-based control techniques. Hierarchical and model-based control approaches have been taken in several studies [3,18,19,20,21,22]. The overall system efficiency is typically described with the conversion efficiency of the reformer unit. High conversion leads to a cost-effective production of hydrogen, with minimized raw material consumption and energy usage. This goal can only be achieved with model-based control approaches, where the operational set-points of fuel processing system are optimized based on the hydrogen demand and conversion efficiency.

This study uses a modular dynamic model for the integrated fuel processing and fuel cell system consisting of ethanol reforming, hydrogen purification in a membrane Water-Gas Shift (WGS) reactor, and a PEM fuel cell stack. This model is used in testing the applicability of a two-layer control hierarchy utilizing an intelligent optimization method. The fuel feed is controlled with a Model Predictive Controller (MPC) in order to minimize the time delay and avoid fuel starvation. The Differential Evolution (DE) algorithm is applied for the upper level control to optimize the steady-state operation point of the system. Hence, the proposed control structure aims to maximize the conversion efficiency with respect to the required level of hydrogen production and constraints. To authors’ best knowledge, this is the first time evolutionary/intelligent optimization is applied for the control of an integrated fuel processing and fuel cell system.

This paper is organized as follows: In Section 2, a simulation model for the integrated fuel processing system is presented, accompanied with the process identification, control, and optimization methods utilized in this study. The process identification results and the comparison of different control strategies is presented in Section 3. Finally, Section 4 contains the discussion and conclusions from this study. 

## 2. Materials and Methods 

The simulated system consists of an integrated process of ethanol steam reforming, followed by WGS separation and a PEM fuel cell stack. The modeling approach and the control structure are described in the following subsections.

### 2.1. Integrated Model of a Fuel Cell System

#### 2.1.1. Steam reforming

The steam reforming is modeled as a Continuous Stirred-Tank Reactor (CSTR) with ideal operation (constant reactor volume, pressure, and temperature). The reaction mechanism adopted utilizes Ni-Al catalyst and is described with two competing reactions in Equations (1) and (2) [23]. The reaction rates are calculated with the kinetic model presented in Equation (3), also adopted from Mas et al. [23].

(1) $$C_{2}H_{5}OH+H_{2}O\to 2CO+4H_{2}$$

(2) $$C_{2}H_{5}OH+3H_{2}O\to 2CO_{2}+6H_{2}$$

(3) $$r_{etOH,j}=k_{0,j}\exp\left[-\left(\frac{E_{a,j}}{R}\right)\left(\frac{1}{T_{R}}-\frac{1}{873}\right)\right]p_{etOH}^{\alpha ,j}$$

Here, *r*_*etOH*,*j*_ is the ethanol conversion in reaction *j* (mol/min·mg), *k*_0,*j*_ is a kinetic constant for reaction *j* (mol/min/mg/atm^α^), *E*_*a*,*j*_ is the activation energy for reaction *j* (J/mol), R is the universal gas constant, *T_R_* is the reactor temperature, *α*,*j* is a stoichiometric constant for reaction *j*, and *p_etOH_* is the partial pressure of ethanol. Therefore, the steam reforming model comprises five components: ethanol, water, hydrogen, carbon dioxide, and carbon monoxide. The material balance for the ethanol is:

(4) $$\frac{dn_{etOH}}{dt}=n_{etOH,in}-n_{etOH,out}-m_{cat}r_{etOH,j}$$

In Equation (4), *n*_*etOH*,*in*_ and *n*_*etOH*,*out*_ are molar flows of ethanol in and out of the reactor (mol/min), *m_cat_* is the mass of the catalyst (mg), and *r*_*etOH*,*j*_ is the reaction rate (mol/min/mg). The material balances for the other components are constructed similarly taking into account the stoichiometric constants in Equations (1) and (2). The kinetic model parameters are listed in Table 1 and the process model parameters in Table 2.

#### 2.1.2. Membrane Water Gas Shift Reactor

The WGS reaction is a reversible reaction aiming to increase the hydrogen content in syngas with the following reaction:

(5) $$CO+H_{2}O↔CO_{2}+H_{2}$$

The model assumes a commercial Cu/ZnO/Al_2_O_3_ catalyst and the volumetric reaction rate *r_wgs_* (mol/cm^3^/s) can be calculated as [24]:

(6) $$r_{wgs}=1.0\times 10^{-3}\frac{\rho_{b}P_{wgs}}{n_{wgs,tot}RT_{wgs}}\exp\left(13.39-\frac{5557}{T_{wgs}}\right)n_{wgs,CO}\cdot \left(1-\frac{n_{wgs,H2}n_{wgs,CO2}}{K_{T}n_{wgs,CO}n_{wgs,H2O}}\right)$$

where *ρ_b_* is the catalyst bulk density (g/cm^3^), *P_wgs_* is the total pressure, *n*_*wgs*,*tot*_ is the total molar flow (mol/s) and *T_wgs_* is the reaction temperature, and *K_T_* is:

(7) $$K_{T}=\exp\left(-4.33+\frac{4577.8}{T_{wgs}}\right)$$

The modeled reactor is a hollow-fiber membrane WGS. The model is adopted from [9,25] with the following assumptions:

(1) the hollow-fibers are CO_2_-selective;
(2) only CO_2_ and H_2_ are able to permeate through the membrane;
(3) system is operated in countercurrent with Argon sweep;
(4) system is isothermal;
(5) there is no axial mixing; and,
(6) the pressure drops on both feed and sweep sides are negligible.

This is basically a distributed parameter model, but here WGS membrane reactor has been modeled as a series of CSTRs. It consists of four interconnected modules: molar balance on the feed side, molar balance on the sweep side, calculation of the permeation flux, and calculation of the reaction rate. The one-dimensional molar balances for the feed side and sweep side are written as Equations (8) and (9), respectively.

(8) $$\frac{dn_{wgs,k}}{dz}=\beta_{k}\frac{1}{4}\pi d_{in}^{2}r_{wgs}-\pi d_{in}J_{k}$$

(9) $$\frac{dn_{shell,k}}{dz}=\pi d_{in}J_{k}$$

where *n*_*wgs*,*k*_ is the molar flow of species *k* in the feed side (mol/s), *β_k_* is the stoichiometric constant according to reaction in Equation (5), *d_in_* is the inner diameter of the hollow fibre (cm), *r_wgs_* is the volumetric reaction rate (mol/cm^3^/s), *J_k_* is the permeation flux (mol/cm^2^/s), and *n*_*shell*,*k*_ is the molar flow in the sweep side (mol/s). 

The permeation flux, *J_k_*, for each gas species *i* is calculated from the equation

(10) $$J_{k}=K_{P,k}\frac{p_{wgs,k}-p_{shell,k}}{d_{mem}}$$

where *K*_*P*,*k*_ is the permeability, *p*_*wgs*,*k*_ and *p*_*shell*,*k*_ are the differential pressures of species *k* in feed and sweep sides, respectively, and *d_mem_* is the membrane thickness (cm). The model parameters are given in Table 2.

#### 2.1.3. PEM Fuel Cell

The ability of a PEM fuel cell to produce electricity is based on catalytic anode and cathode reactions in Equations (11) and (12), respectively.

(11) $$H_{2}\to 2H^{+}+2e^{-}$$

(12) 12O2+2H++2e−→H2O

A simple dynamic model of the fuel cell comprises the electro-chemical behavior as a parameterized, semi-empirical model and molar balances of hydrogen and oxygen in anode and cathode, respectively [26]. The governing equations are adopted from Khan & Iqbal [26] and they are presented in Equations 13–27, with two exceptions; (1) In the electro-chemical model, a term for the concentration overpotential (*η_conc_* in Equation (19)) is added. (2) In the studied integrated fuel processing system, the hydrogen is fed to fuel cell directly from WGS instead of using a hydrogen tank. Therefore, the balance equation for the hydrogen (Equation (13)) deviates slightly from the presentation in [26].

(13) $$\frac{V_{a}}{RT}\frac{dp_{H2}}{dt}=n_{H2,in}-n_{H2,out}-n_{H2,consumed}$$

(14) $$\frac{V_{c}}{RT}\frac{dp_{O2}}{dt}=n_{O2,in}-n_{O2,out}-n_{O2,consumed}$$

(15) $$\frac{dV_{act}}{dt}=\frac{i}{C_{dl}}-\frac{V_{act}}{R_{act}C_{dl}}$$

(16) $$n_{H2,consumed}=\frac{n_{fc}i}{2F}$$

(17) $$n_{O2,consumed}=\frac{1}{2}\frac{n_{fc}i}{2F}$$

(18) $$n_{O2,out}=k_{C}\left(p_{O2}-p_{bpr}\right)$$

(19) $$V_{fc}=n_{fc}\cdot \left(E_{0}-\eta_{act}-\eta_{ohm}-\eta_{conc}\right)$$

(20) $$E_{0}=1.229-0.85\times 10^{-3}\left(T-298.15\right)+4.3085\times 10^{-5}T\ln\left(p_{H2}p_{O2}^{0.5}\right)$$

(21) $$\eta_{act}=-0.948+T\left(0.00286+0.0002\ln(A_{a})+4.3\times 10^{-5}\ln(c_{H2})\right)+7.6\times 10^{-5}T\ln(c_{O2})-1.93\times 10^{-4}T\ln(i)$$

(22) ηohm=181.6⋅[1+0.03(iAa)+0.062(T303)2(iAa)2.5]12.5−0.634−3(iAa)⋅exp[4.18(T−303T)]dmeaAai

(23) $$\eta_{conc}=-\frac{RT}{2F}\ln\left(1-\frac{i}{i_{\max}}\right)$$

(24) $$c_{O2}=p_{O2}\cdot 1.97\times 10^{-7}\exp(\frac{498}{T})$$

(25) $$c_{H2}=p_{H2}\cdot 9.174\times 10^{-7}\exp(\frac{-77}{T})$$

(26) $$V_{act}=-\eta_{act}$$

(27) $$R_{act}=\frac{V_{act}}{i}$$

In equations above, *V_fc_* is the output voltage of single cell, *i* is the cell current, *E_Nernst_* is the thermodynamic potential, *η_act_*, *η_ohm_* and *η_conc_* describe the activation, Ohmic, and concentration overpotential of the fuel cell, respectively. The fuel cell specific model parameters (*V_c_*, *V_a_*, *C_dl_*, *p_pbr_*, *k_C_*, *A_a_*, *d_mea_*, *i_max_*) are given in Table 2. The fuel consumption that is described in Equations (16) and (17) is dependent on *n_fc_*, the number of cells in the fuel cell system.

### 2.2. Control Framework

In the simulated fuel processing system, the control objective is to stabilize the fuel cell power output with fast response to load changes. However, the system shows large delays between the inputs and outputs which stem from the complexity of the process, and especially from the slow dynamics of the hydrogen purification stage in the WGS reactor. From the control point of view, it then forms a stiff system having a reactor with slower dynamics compared to other two process stages. Thus, the control strategy should focus on compensating for the long delay time with predictive model-based methods.

**Figure 1:**
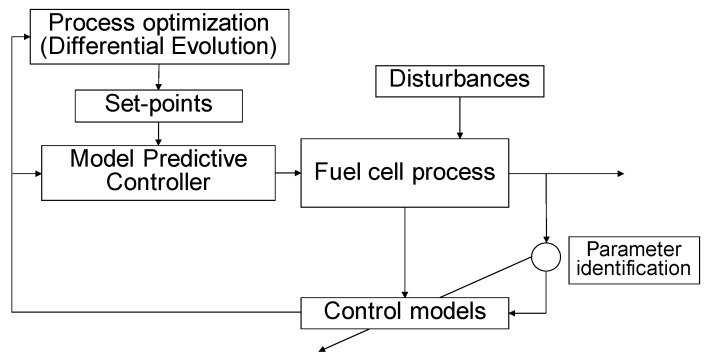
A control framework for the simulated fuel cell process.

The proposed method involves two-layer control hierarchy presented in Figure 1. The control framework includes a base layer controller that is facilitated by PI-controller or MPC and a higher level intelligent optimizer established with Differential Evolution. The MPC is here constructed as a SISO system or an alternative MIMO system with linear autoregressive models. In the former case, the MPC utilizes the desired cell current (and therefore the desired hydrogen feed flow to the fuel cell) as a reference signal, the hydrogen molar flow to the fuel cell as a measured output, and the steam reforming feed W/E-ratio as a manipulated variable. In the latter case, the total molar feed flow to steam reforming is manipulated as well. A hierarchical optimization approach can be adopted in order to maximize the conversion efficiency in the reformer unit. The higher level optimizer provides optimal set-points for the lower lever controller. The process optimization is based on the DE algorithm utilizing predictive control model of the studied process. The optimizer was launched every time when the hydrogen set-point is changed, and a new steady state was reached. After the optimization routine, the maximum conversion efficiency found was introduced as a set-point to the MPC.

#### 2.2.1. Model Predictive Control

MPC utilizes an identified process model to predict and optimize the behavior of the controlled system according to some cost function [27]. In this study, Matlab’s built-in Model Predictive Control Toolbox is utilized. The empirical process models (control models) are identified from the open loop simulations with the integrated model of fuel cell system.

#### 2.2.2. Model identification

The process dynamics for the MPC design are here described with linear input-output models. A general structure for a linear model is:

(28) A(q)y(t)=B(q)u(t)+C(q)e(t)

where *A*(*q*) denotes the parameters of *q*-1 output delays, *B*(*q*) is the same for inputs *u*(*t*), and *C*(*q*) for the approximated unmeasured disturbance *e*(*t*). The model identification is performed with Matlab’s built-in System Identification Toolbox, utilizing the prediction error method and the input-output data obtained from the integrated simulation model that is described in Section 2.1.

#### 2.2.3. Differential Evolution

DE is an optimization method with basis in evolution theory [28]. The DE algorithm has several preferable properties; it is a global optimizer, which can be configured to avoid local minima. The algorithm operates with floating-point numbers (real values), making the optimization simple and fast. Also, the number of design parameters is low—the parameters to be set are related to the population size, minimum and maximum allowed initial values of the population members and crossover probability. The main difference between the DE and genetic algorithms is in the mutation operation. In genetic algorithms, the mutation is randomly performed, whereas DE uses the differences of chromosomes and their arithmetical combinations to generate new population members. The parameters that were used in this study are presented in Table 3. Here, the implementation is performed with a custom-made Matlab function utilizing the DE variant with the best base vector selection for mutation, one difference vector and exponential crossover (DE/best/1/exp).

#### 2.2.4. Objective Function

Two manipulated variables, namely the W/E-ratio and the total molar flow to reformer, were selected for optimization. Also, two outputs, the conversion efficiency and the hydrogen flow rate after the WGS reactor were included in the analysis. The general form of the cost function to be minimized in this case was

(29) $$CF=\min\left\{h_{1}\cdot \left(100-y_{1,SS}\right)+h_{2}-u_{1}(k)+h_{3}u_{2}(k)+h_{4}\cdot \left|r_{2}(k)-y_{2,SS}\right|\right\}$$

where constants *h*_1–4_ are the tuning parameters, *u*_1_(*k*) and *u*_2_(*k*) are the manipulated W/E-ratio and the total molar flow at time instant *k*, respectively, *y*_1,*SS*_ and *y*_2,*SS*_ are the steady state values of the conversion efficiency and hydrogen flow rate after an input change, and *r*_2_(*k*) is the set-point for the hydrogen flow rate.

The steady state equals to the estimated future value after 200 s. The cost function is designed to penalize deviations from the set-point and to favor high conversion. In addition, the low total flow and high W/E-ratio are preferable, and therefore they minimize the cost function. The tuning parameters *h*_1–4_ utilized were 1, 5, 50, 100, respectively.

## 3. Results

**Figure 2:**
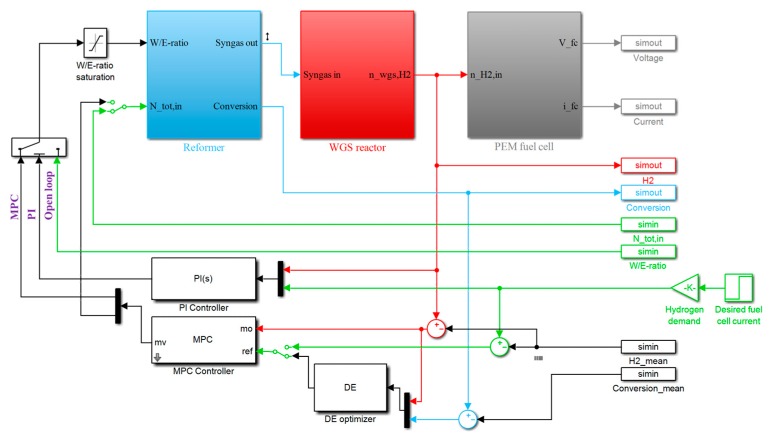
The simulation model. On top of the figure, the three process models are masked into the respective subsystems. On right hand side of the figure, the data communication between Simulink and Matlab workspace is established. The different control options are depicted in bottom of the figure, and finally the selection between the open loop, Proportional-Integral (PI) control, and Model Predictive Controller (MPC) operation can be made on the left hand side of figure.

The simulation model and the control framework presented above were formulated into a Matlab/Simulink® model illustrated in Figure 2. First, the control models were identified with open loop simulations, followed by the comparison of the base control strategies, and finally the hierarchical control strategy was tested. The results are presented in the following subsections.

### 3.1. Identification of the Control Models

**Figure 3:**
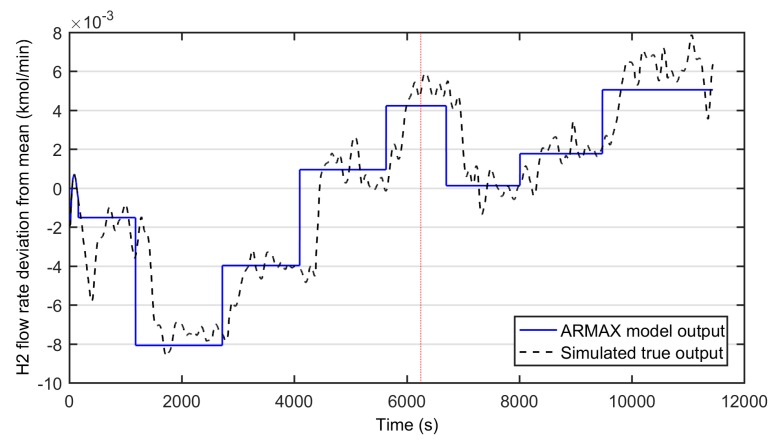
Modeling results of the autoregressive moving average model with exogenous input (ARMAX)-model for the water-ethanol ratio as input and hydrogen flow rate as modeled output (mean removed). Solid line: the modeled hydrogen flow rate, dashed line: simulated true output, dotted line: division of training (on right) and testing data (on left).

First, the hydrogen flow rate was approximated with an autoregressive moving average model with exogenous input (ARMAX). The W/E-ratio was used as a model input based on its strong effect on hydrogen production [29]. The magnitudes of both the input and output was synchronized by removing the mean values from the model variables. Figure 3 depicts the identification results. The model parameters were selected based on the fit index describing the percentage of the output variations that is reproduced by the model over the training and testing data. In this case, the fit index was 57.13. The resulted model parameters are presented in Equation (30).

(30) $$\begin{array}{l} A(q)=1\\ B(q)=-0.005027q^{-158}-0.0002274q^{-159} \end{array}$$

The resulted model has no poles and it includes two delay terms at time lags of 158 and 159 s. Thus, according to the model, it seems that simulated process exhibits large dead time and delay from input to output. 

**Figure 4:**
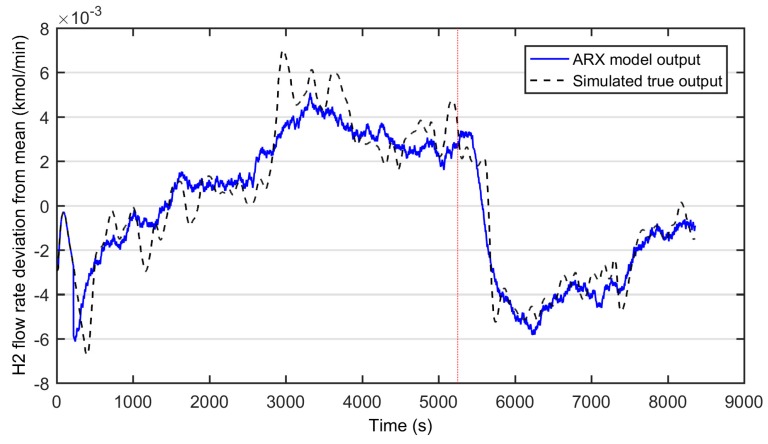
Identification results of the autoregressive model with exogenous input (ARX)-model with two inputs: the water-ethanol ratio and total feed to reformer unit. Solid line: the modeled hydrogen flow rate, dashed line: simulated true output, dotted line: division of training (on right) and testing data (on left).

The second model candidate used two inputs; the W/E-ratio and the total molar flow to reformer. The selected model structure was an autoregressive model with exogenous input (ARX). In this case, model parameter identification resulted in a model with one pole (one lagged output as an input), together with two other inputs both with delays of 220 s. The model identification results in this case are presented in Figure 4 and the model parameters are given in (31).

(31) $$\begin{array}{l} A(q)=1-0.996q^{-1}\\ B(q)=\left[\begin{array}{c} -3.549\times 10^{-5}q^{-220}\\ -0.008549q^{-220} \end{array}\right] \end{array}$$

According to Figure 4, the ARX-model with two input variables can smoothly follow the simulated true output. The fit index in this case was 67.67, outperforming the ARMAX model (57.13) with a moderate cost of calculation power. However, the more critical model property was its ability to capture the process dynamics; in preliminary testing, the ARX-model lead to an unstable controller behavior, whereas the simpler ARMAX-model seemed to capture the process dynamics better.

**Figure 5:**
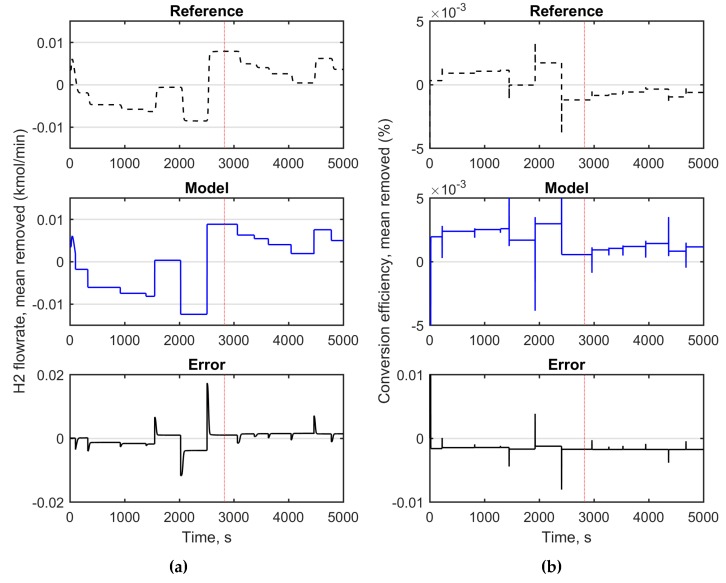
Simulated model outputs of multiple-input multiple-output ARX-model. (**a**): hydrogen flow rate, (**b**): conversion efficiency. Solid line: model outputs, dashed line: simulated true value, dotted line: division of training (on right), and testing data (on left).

A control model was also needed in this application for the MPC controller and for cost function calculation during optimization. For this purpose, an open loop simulation was performed, where the two manipulated variables (W/E-ratio and total molar flow to reformer) were randomly changed and the response in hydrogen flow rate and conversion were recorded. As in the former cases, the mean values of the model variables were removed prior to identification. The modeling results are depicted in Figure 5. The best performing model was found to be an ARX-model with eight free parameters and 100 s delays:

(32) $$\left[\begin{array}{c} y_{1}(t)\\ y_{2}(t) \end{array}\right]=\left[\begin{array}{c} B1(q)\\ B2(q) \end{array}\right]\left[\begin{array}{c} u_{1}(t)\\ u_{2}(t) \end{array}\right]+C(q)\left[\begin{array}{c} e_{1}(t)\\ e_{2}(t) \end{array}\right]\;\begin{array}{l} B1(q)=\left[\begin{array}{cc} -0.003q^{-100} & -0.0082q^{-101}\\ -0.0235q^{-100} & 1.1938q^{-101} \end{array}\right]\\ B2(q)=\left[\begin{array}{cc} 0.002231q^{-1} & -0.001066q^{-2}\\ -0.2112q^{-1} & 0.08326q^{-2} \end{array}\right] \end{array}$$

The fit index for the modeled hydrogen flow rate (*y*_1_(*t*)) with training and testing data was 53.4 and for conversion (*y*_2_(*t*)) 21.2. According to the results in Figure 5, the conversion values are underestimated but with an almost constant bias. On the other hand, the model captures the direction and magnitude of the changes. Therefore, the model should be able to provide a feasible estimate for prediction and optimization purposes.

### 3.2. Comparison of PI and MPC

The performance of a MPC as a base level controller was tested and the results were compared to conventional PI control. A SISO system was concerned with the hydrogen consumption (based on the FC current, see Equation (16)) as a reference, the measured hydrogen flow from the WGS reactor as a controlled variable, and the reformer water-ethanol ratio as a manipulated variable.

The optimal tuning parameters for the MPC were as follows; control interval (1 s), prediction horizon (400 s), control horizon (1 s), input rate weight (0.05), output weight (1), and the magnitude gain of the input disturbance model (0.001). Input and output constraints of the MPC controller were set so that any non-ideal behavior of the controller could be observed without restriction from the optimizer algorithm. For MPC simulation, the removed mean value in model identification (0.04 kmol/min) was added for scaling the control model to the actual output values. The PI controller had a proportional gain of −9.46 and an integral gain of −0.50.

**Figure 6:**
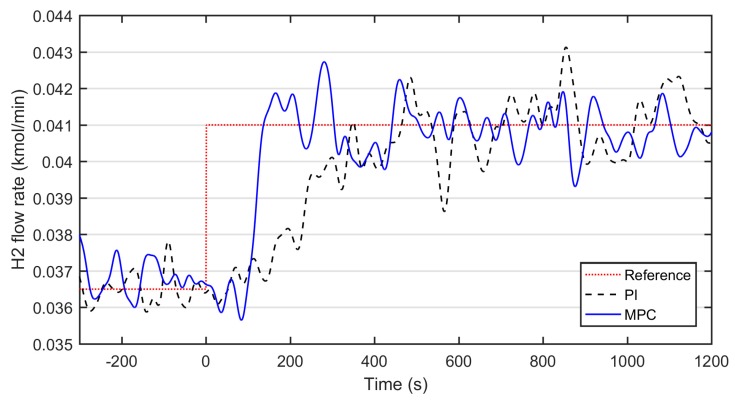
Simulated control results of the MPC and PI controller during a step change in the required hydrogen flow rate.

Both PI and MPC controllers were then run with the same random seed of the noise source and the stepwise 12% increase of hydrogen consumption (from 0.0365 to 0.041 kmol/min) in the simulation. The observed hydrogen flow rates with both controllers are presented in Figure 6.

According to Figure 6, a new steady-state with a variation of ± 0.0015 kmol/min (4% of the set-point value), is reached after 900 s with the PI. The rise time (50%) was approximately 300 s. In comparison, MPC controller has a rise time of 150 s and it reaches the set-point only in 200 s with a variation range of ± 0.001 kmol/min (2.4% of the set-point value). Hence, MPC shows a better performance, both in terms of the reduction of process variation and dynamics.

### 3.3. MPC with a Higher Lever Optimizer

**Figure 7:**
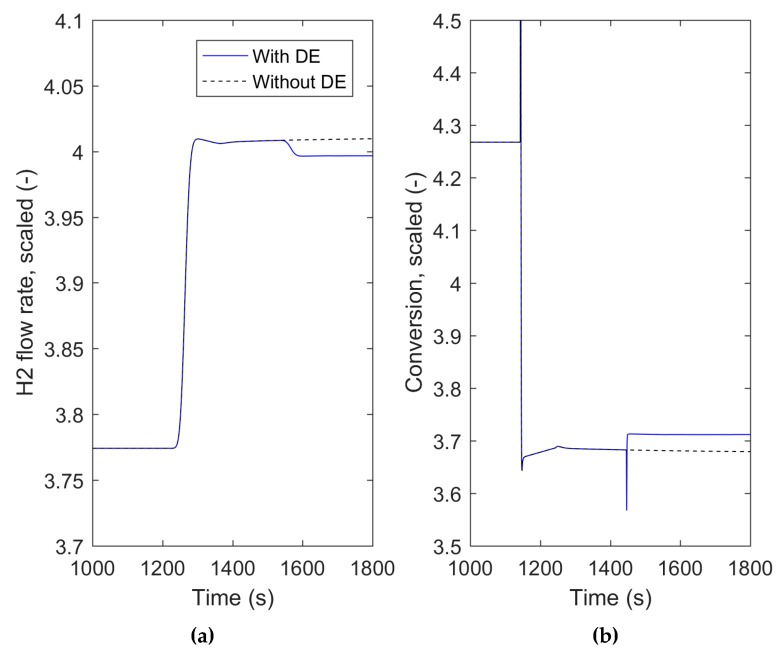
The hydrogen flow rate (**a**) and the conversion (**b**) behavior with and without the higher level optimization (DE). Step change to the hydrogen flow set-point at 1100 s. New optimized conversion value introduced to MPC at 1400 s.

The optimization capability of the presented method was demonstrated by simulating the integrated fuel processing model and the controller, with a step change in the hydrogen flow rate at time instance 1100 s. After the new steady state at time instance 1400 s, the higher level optimizer was launched to calculate the maximum reachable conversion efficiency for the current state. This value was further applied to MPC as a set-point. The simulation results are presented in Figure 7, where the values of the hydrogen flow rate and conversion efficiency have been scaled to the same magnitude. 

According to the results in Figure 7, the higher level optimizer resulted in MPC increasing the W/E-ratio, which was eventually seen as a 2% increment in the conversion efficiency. The hydrogen flow rate was kept within the limits of ± 1.25% from the set-point. However, a spike can be observed due to the fast process dynamics related to conversion calculation in the process model. The simulation was repeated without the optimizer (dashed line Figure 7). In this case, the step change in the hydrogen flow rate results in a slight decrease in the conversion efficiency.

## 4. Discussion and Conclusions

A simulation model for an integrated fuel processing system consisting of ethanol reformer, WGS membrane reactor, and PEM fuel cell was developed. The presented model was then applied for process control and optimization. For this purpose, empirical input-output models were developed from the simulation data and then utilized in a model-based control framework.

It was found that an ARMAX-model for the hydrogen flow rate was sufficient to capture the delay dynamics of the process and the magnitude of the changes when the W/E-ratio was altered. It was also indicated during process identification that the dynamics of the integrated system varied depending on the processing stage. This kind of a stiff system motivated the application of MPC method.

Indeed, the traditional PI controller was outperformed by MPC in the simulations where the set-point of the hydrogen flow rate was changed stepwise. By applying MPC, a reduction of approximately 30% in process variation was achieved in comparison to PI. The settling time with MPC was 200 s compared to that of 900 s with the PI controller.

The operation of the most critical part of the simulated process chain, namely production of hydrogen in the reformer, was further optimized by combining a DE algorithm with MPC and forming a two-level hierarchical control system. A case example was presented, where the optimization framework was applied to maximize the conversion efficiency in the reformer. In simulations, the optimized conversion was 2% higher without violating the required hydrogen flow rate. The hierarchical control system, where the higher level optimizer calculated the set-point to an MPC, has a potential to reduce the consumption of raw materials used for energy production, namely the ethanol feed.

The following conclusions can be drawn based on the simulation results:

(1) In the integrated fuel processing system, the hydrogen production and purification steps play a major role in process dynamics. From the control point of view, these are more critical parts than the PEM fuel cell with fast dynamics.
(2) Load changes in the hydrogen flow require a long settling time. This property limits the applicability of the integrated fuel cell process.
(3) The empirical linear models are suitable to describe the simulated hydrogen production process in the tested operating range. Such models may simplify and increase the robustness of the model-based control system in a real-world application.
(4) Hierarchical control strategy can simultaneously affect the economy of the hydrogen production and the regulation of the flow rate. The increased conversion efficiency can potentially be achieved with a small control effort.

The control framework and the conversion efficiency optimization of the reformer were based on the manipulated variables that most influenced on process outputs. Alternatively, the selection of variables could be based on controllability analysis; for example, in Pravin et al. [30], the pairings for the single-loop controllers in an integrated fuel processing system were based on the Relative Gain Array method. The simulations in this study involved a limited range of operating conditions. It should be noted that the influence of other adjustable model parameters in the integrated fuel processing system could be profound in different operating conditions. This may influence the straight utilization of the results and conclusions. In addition, it would be interesting to study the optimization of similar energy conversion systems with varying fuel types. By taking advantage of the recent modeling efforts, the control framework presented could also be extended to integrated systems consisting, for instance, an autothermal reforming [30] or microchannel reactors [31].

## Figures and Tables

**Table 1:** Kinetic model parameters for ethanol reforming [23].

Parameter	Reaction (1)	Reaction (2)
*k*_o_ (mol/min/mg/atm)	5.74 × 10^−4^	1.85 × 10^−4^
*E*_a_ (J/mol)	1.44 × 10^5^	2.07 × 10^5^
α (-)	0.75	0.8

**Table 2:** Simulation parameters.

Parameter	Value	Comment
*N*_*tot*,*in*_	0.0457	Reformer nominal input molar flow rate, mol/min
*T_R_*	898	Reformer temperature, K
*P_R_*	3	Reformer pressure, atm
*W/E*	0.1–0.25	Reformer nominal input water/ethanol-ratio, -
*m_cat_*	11400	Mass of catalyst in reformer, mg
*n_m_*	5000	Number of membrane fibres, -
*N_sweep_*	1.6698 × 10^−5^	WGS shell side sweep gas molar flow rate, mol/s
*T_wgs_*	453	WGS temperature, K
*P_wgs_*	303975	WGS tube side pressure, N/m^2^
*ρ_b_*	1395	WGS catalyst bulk density, kg/m^3^
*R*	8.314	Universal gas constant, J/Kmol
*V_wgs_*	4.7909 × 10^−7^	WGS total volume, m^3^
*n_wgs_*	120	Number of CSTR elements in WGS, -
*P_s_*	101325	WGS shell side pressure, N/m^2^
*K*_*p*,*CO*2_	1.3392 × 10^−12^	^1^ WGS CO_2_ permeability, mol m/m^2^s Pa
*K*_*p*,*H*2_	3.3480 × 10^−14^	^2^ WGS H_2_ permeability, mol m/m^2^s Pa
*d_mem_*	5 × 10^−6^	WGS membrane thickness, m
*d*	1× 10^−3^	WGS inner diameter of fibre, m
*l*	0.61	WGS length of fibre, m
*n*_*O*2,*in*_	0.0177	FC oxygen feed, mol/s
*F*	96,487	Faraday constant, s A/mol
*n_fc_*	35	FC number of cells, -
*T_fc_*	345	FC temperature, K
*V_c_*	0.01	FC cathode volume, m^3^
*V_a_*	0.005	FC anode volume, m^3^
*P_bpr_*	3	FC oxygen pressure at the outlet, atm
*k_c_*	0.065	FC cathode flow constant, mol/s atm
*A_a_*	232	FC anode active area, cm^2^
*d_mea_*	0.0178	FC membrane thickness, cm
*C_dl_*	8.12	FC equivalent capacitance, F
*i_max_*	300.1	FC limiting current, A

^1^ Equals to 4000 barrels, ^2^ Equals to 100 barrels.

**Table 3:** Algorithm parameters for Differential Evolution (DE).

Convergence Criterion	Initial Population Bounds	Population Size	DE Step Size	Crossover Probability
*CF* ≤ 10^−6^ or *iter_max_* = 500 is reached	W/E = [2.5 9] *N*_*tot*,*in*_ = [0.01 0.09]	20	0.2	0.4
